# National Non-Communicable Diseases Conferences- A Platform to Inform Policies and Practices in Tanzania

**DOI:** 10.5334/aogh.4112

**Published:** 2024-03-04

**Authors:** Davis E. Amani, Harrieth P. Ndumwa, Jackline E. Ngowi, Belinda J. Njiro, Castory Munishi, Erick A. Mboya, Doreen Mloka, Amani I. Kikula, Emmanuel Balandya, Paschal Ruggajo, Anna T. Kessy, Emilia Kitambala, Ntuli Kapologwe, James T. Kengia, James Kiologwe, Omary Ubuguyu, Bakari Salum, Appolinary Kamuhabwa, Kaushik Ramaiya, Bruno F. Sunguya

**Affiliations:** 1 Muhimbili University of Health and Allied Sciences, 9 United Nations Road, Upanga West P O Box 65001, Dar es salaam, Tanzania; 2 Ministry of Health, P O Box 743 Dodoma, Tanzania; 3 President’s Office Regional Administration and Local Government, P O Box 1923 Dodoma, Tanzania; 4 Tanzania Non-Communicable Diseases Alliance, P O Box 65201 Dar es salaam, Tanzania; 5 Tanzania Diabetes Association, P O Box 65201 Dar es salaam, Tanzania; 6 Shree Hindu Mandal Hospital, P O Box 581 Dar es salaam, Tanzania

**Keywords:** non-communicable diseases (NCDS), NCDs conferences, NCDs policies, Tanzania

## Abstract

**Background:**

Non-communicable diseases (NCDs) arise from diverse risk factors with differences in the contexts and variabilities in regions and countries. Addressing such a complex challenge requires local evidence. Tanzania has been convening stakeholders every year to disseminate and discuss scientific evidence, policies, and implementation gaps, to inform policy makers in NCDs responses. **This paper documents these dissemination efforts and how they have influenced NCDs response and landscape in Tanzania and the region**.

**Methods:**

Desk review was conducted through available MOH and conference organizers’ documents. It had both quantitative and qualitative data. The review included reports of the four NCDs conferences, conference organization, and conduct processes. In addition, themes of the conferences, submitted abstracts, and presentations were reviewed. Narrative synthesis was conducted to address the objectives. Recommendations emanated from the conference and policy uptake were reviewed and discussed to determine the impact of the dissemination.

**Findings:**

Since 2019, four theme-specific conferences were organized. This report includes evidence from four conferences. The conferences convened researchers and scientists from research and training institutions, implementers, government agencies, and legislators in Tanzania and other countries within and outside Africa. Four hundred and thirty-five abstracts were presented covering 14 sub-themes on health system improvements, financing, governance, prevention intervention, and the role of innovation and technology. The conferences have had a positive effect on governments’ response to NCDs, including health care financing, NCDs research agenda, and universal health coverage.

**Conclusion:**

The National NCDs conferences have provided suitable platforms where stakeholders can share, discuss, and recommend vital strategies for addressing the burden of NCDs through informing policies and practices. Ensuring the engagement of the right stakeholders, as well as the uptake and utilization of the recommendations from these platforms, remains crucial for addressing the observed epidemiological transition in Tanzania and other countries with similar contexts.

## Background

The United Nations Sustainable Development Goal’s (SDG) target 3·4 calls for all member states to reduce premature mortality from NCDs by one third by 2030 through prevention and treatment, as well as to promote mental health and well-being [1]. Despite the efforts in advocacy and investments made thus far, most of low-income and middle-income countries (LMICs) are off track to reach this target [2,3]. Moreover, three in four NCDs deaths are in LMICs, translating to 41 million deaths annually [3]. The current epidemiological transition in these countries–which are already overburdened by other health challenges–further strain the already weakened health systems [4].

Sustaining efforts against NCDs call for a multisectoral collaboration and cooperation at national, regional, and global levels [5]. Evidence suggests an alarming increase of NCDs in LMICs requires coordinated national and global responses [6]. Stakeholders’ engagement is recommended across sectors beyond health [5,7,8]. To this end, the Tanzanian National NCD prevention and control program collaborates with local, regional, and international stakeholders in the development and implementation of NCDs policies [9,10].

In strengthening the advocacy efforts for NCDs, Tanzania’s government launched the National NCDs week in 2019. One of the activities during this week is the National NCDs scientific conference. This conference has served as a platform for various stakeholders to share, analyze, and discuss existing local evidence, activities, and strategies, and provide recommendations for NCDs response. The conference brings together an average of 500 people with various backgrounds and roles in addressing the NCDs annually. They include government leaders, policy makers, NCDs research community, healthcare professionals, development, and implementing partners. Attendees are from local and international organizations, non-governmental organizations, and professional associations. Others are university students and representatives from secondary schools who are specially invited to raise awareness among the scientists of the next generation. The Ministry of Health in Tanzania uses the National NCDs conference to promote NCDs research dissemination, establish, engage, and sustain partnership and strategies for incorporating the evidence for policies and plans.

The national NCDs conferences have been successfully conducted for five years, but evidence of its impact has not been systematically presented for other countries with similar context to adopt. This review provides evidence on how these conferences were organized, evidence presented, and its roles in NCDs response. Lessons and challenges through its implementation may be useful for the countries contemplating to initiate similar platform.

## Methods

### Design and context

Desk review was conducted to examine and document the initiation, organization, outcomes, and policy implication of National NCDs Conference in the NCDs response in Tanzania. Most of both quantitative and qualitative data reviewed were from the Ministry of Health, conference proceedings and reports, and recommendations’ reports. The review period stretched from the inception of NCDs week in 2019 through the fourth NCDs conference in 2022. Five conferences have been held so far, but the last available report was of the fourth conference by the time of writing this manuscript.

### Review process and data abstraction

This policy paper documents the lessons learned from the organization of the NCDs conferences. The data used was extracted from the conference reports and conference raw documents and materials such timetables, policy briefs, abstract books, and conference website data. Available online registration forms were retrieved and reviewed to establish groups and diversity of stakeholders who participated in the conference. From these documents, abstracted information included, the number and type of stakeholders (national and international) engaged, relevance of topics presented and discussed, gaps identified, and challenges experienced. Both qualitative and quantitative data were extracted.

### Analysis and presentation of data

Quantitative data was analyzed descriptively to generate descriptive statistics and figures. Regarding the non-quantitative information including conference themes and subthemes and conference recommendations, summaries were generated and presented in form of tables to enrich discussion thereof.

### Ethical consideration

The ethical approval was not required for this study since it was not a study on human subjects but rather a documentation of lessons learned from organizing the Tanzania NCDs conferences. All the authors were members of the conference organization committee and hence had access and permission to use the conference data and materials from the conference chairperson.

## Findings

### Rationale for the conference

The National NCD conferences have been organized annually and bring various stakeholders and individuals working in addressing NCDs in Tanzania to learn, discuss thoughts, network, and share ideas to influence policies and practices. Stakeholders were from different governmental organizations such as the central government, members of parliament, and local government authorities. Participants were also from non-governmental organizations provided they were interested in or working to address NCDs.

The number of attendees and stakeholders has been increasing steadily from 200 participants in the inception conference in November 2019 to 780 participants in the fourth conference conducted in November 2022. To increase participation and mitigate the challenges brought by COVID-19, the third and fourth National NCDs conferences allowed virtual participation. Overall, the National NCDs conference has been an important step towards addressing NCDs in Tanzania. Moreover, it has brought a special forum for evidence based and innovative ways to address NCDs. The platform has been a tool for raising attention towards NCDs among policy makers, researchers, healthcare providers, and community members, and it helped to push the national NCDs agenda.

### Conference preparation and stakeholder involvement

Conference organization has been done by the conference organizing committee (CoC) made of three sub-committees. They are financial, scientific, and logistics subcommittees. The latter was made of procurement, venue preparation, invitation, and ICT subunits. Each subcommittee consisted of members from the central and local government, private and public health facilities, research and training institutions, and Non-Governmental Organizations (NGOs). In total, the committee was composed of a minimum of 40 members with diverse knowledge, skills, and experience.

Committee members from government authorities included representatives from the Ministry of Health (MoH), President’s Office, Regional Administration and Local Government (PORALG), Ministry of Foreign Affairs (MoFA), and regional and council health management teams (RHMTs and CHMTs). Ten institutions and organizations also constituted the CoC, seven of which were government-owned. Planning for the next conference starts at the conclusion of the current one, when the region for the upcoming conference is selected. Starting August in the year of the conference, posters are prepared and disseminated out to call for abstracts and participation. The preparations become intensive at the end of September until November where the actual conference is held for the given year. At the end of the conference, participants are asked to rate the conference through online anonymous evaluation survey. In 2021 for example, 100 people participated in the survey, where 98% (n = 98) were satisfied with the overall conference organization and conduct, and similarly 98% (n = 98) were satisfied with the knowledge they had gained by attending the conference **(Supplementary Figure 1)**.

The number and diversity of stakeholders who took part in the conference also differed over the three years where reports were available. In 2020, 2021, and 2022, the number of personnel who attended the conferences was 500, over 600 (20% increase) and 780 (56% increase), respectively. Reports also showed an increase in involvement of stakeholders from different sectors, both from Tanzania and the international community such as civil society organizations and the World Diabetes Foundation. In the most recent conference, participants came from 17 different countries **(Supplementary Table 1)**.

In these events, there was also involvement of secondary school students who were specially invited to build enthusiasm for NCDs response and awareness; law enforcement officers such as the police force and traffic police, who are implementers and first responders in road traffic injuries; patients and patients’ organizations, as they are important recipients of services; and the local community members from where the conference was being held.

### Resource mobilization and budget

Fundraising activities were spearheaded by the finance subcommittee. The subcommittee was responsible for developing the budget for all conference activities, developing and implementing strategies for soliciting funding from different stakeholders, managing the disbursement of funds based on budget items, and developing a financial report of the conference. The sources of funds for the conference have largely come from voluntary sponsorships, government allocation, exhibition charges, and participation fees. Support for the conference has been increasing over the years. For example, the budget and funding increased by 99.5% from TZS 103,620,380 in 2020 to 209,680,348 in 2022.

The contribution from sponsors varied yearly. Since 2020, one fifth to nearly a third of the conference budget was accounted for by the Government of Tanzania through the Ministry of Health. This indicates the commitment of the government in efforts geared to address the burden of NCDs in the country. Other sources constitute revenues from the registration of individuals and institutions, as well as rented exhibition booths. On average, these accounted for a quarter of the conference budget. For the past four years, World Diabetes Foundation through the Tanzania Diabetes Association and Tanzania Non-Communicable Diseases Alliances (TDA/TANCDA) accounted for 100% of the budget contributions from the NGOs. On the other hand, UNICEF was the sole UN organization and development partner who supported the conference **(Supplementary Figure 2)**.

### Abstracts presented by themes

Abstracts and topics presented in the conferences, discussions, and recommendations which emanated from them were guided by central conference themes. Since 2020, a total of 435 abstract presentations were made, half of which came from college students. Each year had a different theme that considered the contemporary challenges and was developed through a consultative and consensus process. Table 1 below highlights the themes and the number of abstracts presented for all the past four conferences.

**Table 1:** Conference themes.


	2019	2020	2021	2022

**Theme**	Multisectoral Engagement in Prevention, Care and Management of NCDs in Tanzania	Strengthening Health Systems to prevent and Control Non-Communicable Diseases in Tanzania	Multisectoral Engagement and Collaboration in addressing Non-Communicable Diseases in Tanzania	Enhancing Scalability and Sustainability of Preventive and Integrated, Accessible, Quality Non-Communicable Diseases Care in Tanzania

**Subthemes**		Health System strengthening in addressing the burden of NCDs in Tanzania NCD and Comorbidities in Tanzania Prevention and Promotion Risk Factors for NCDs Care and Treatment of NCDs NCDs Governance Research, Innovation and Technologies	Governance, Role of Public Private Partnership, Civil Societies and Non-Health Sectors. Health System Strengthening, NCD Financing and Universal Health Coverage. Risk factors and Social Determinants of NCDs NCD Prevention and Health Promotion. NCD Care, Treatment and Comorbidities. Research, Innovation and Technology.	Risk factors and burden of NCDs and related comorbidities; focus for advocacy prevention and stakeholders’ engagement strategies. Sustainable and scalable integrated care and treatment approaches for NCDs and their related comorbidities Technology and innovative approaches on prevention and treatment of NCDs Mental health hereditary and degenerative diseases; prevention treatment and control

**Number of Abstracts**		**140**	**179**	**116**


In ensuring adequate and exhaustive focus on components pertinent in addressing the burden of NCDs, subtopics/subthemes were constructed. These reflected of such areas as health systems, NCDs prevention, treatment and care, health promotion, NCDs burden and risk factors, and stakeholders’ engagement. The conference abstracts were released in a special issue on the Tanzania Medical Journal (TMJ) for both 2020 and 2021 [11,12].

### Conference Recommendations and Influence

At the end of the conference recommendations were generated and channeled to the responsible organization for action based on the conference’s presentations, panel discussions, and meetings. Most of the recommendations were directed to the government of Tanzania through the Ministry of Health for the purpose of developing, revising, and implementing an appropriate response against the rising burden of NCDs in the country. In these conferences, all closing ceremonies were graced by the permanent secretary of Ministry of Health, or representative thereof, who is the chief implementer of the NCDs response. Table 2 provides a summary of recommendations generated from the four conferences.

**Table 2:** A summary of conference recommendations.


RECOMMENDATIONS FROM THE 2020 NCDS CONFERENCE	RECOMMENDATIONS FROM THE 2021 NCDS CONFERENCE	RECOMMENDATIONS FROM THE 2022 NCDS CONFERENCE

Develop and implement Social Behavioural Change and communication strategies for NCDs. Ensure the effective implementation of the existing NCDs strategic plan and align it with other relevant sectorial strategies. Engage learning institutions from early childhood to higher learning to realize sustained behavioural change for NCDs prevention. Mainstream and evaluate the role and implementation of NCDs education from primary education to higher learning institutions. Form a strong multi- and intersectoral coordination mechanism from national level down to the community level. Ensure coverage of health insurance to cover all Tanzanians and reduce payments for health care. The current efforts for universal health coverage through mandatory health insurance are commendable and need to be fast-tracked. Strengthen competencies of the existing human resources for health to prevent and control NCDs. Ensure protection of public spaces for physical and recreational activities.	Strengthen the strategies to enhance and ensure universal health coverage (UHC). Liaise with other stakeholders in facilitating the integration of services thus harmonizing the efficient utilization of the existing resources. Facilitate capacity-building among healthcare workers. Revise policies and strategies on resource mobilization and allocation. Consider priority setting reflective of needs of the population, e.g., the elderly, children, and those in remote areas. Improve the existing insurance schemes to include most services and render them accessible to all. Explore and consider innovative financing for NCDs, including imposing levies on cigarette companies. Use disease registries to track the trends of diseases and their related outcomes. Emphasize and promote health-seeking behaviours	Leverage the potential of new technologies to improve training, and establish and implement new, effective, and acceptable NCDs prevention and treatment services. Strengthen research, training, and healthcare infrastructures to foster generation and uptake of local evidence and innovation solutions. Explore and introduce alternative and effective financing models for Integrated NCDs treatment and care services. Promote and ensure equity and equality in accessing NCDs services. Foster and sustain community engagement and multisectoral collaborations in addressing the burden of NCDs. Explore, evaluate, develop, and implement a resilient and responsive NCDs referral system. Improve, scale-up, and sustain NCDs screening services. Promote cost-effective, community-based NCDs prevention services. Leverage on existing resources and opportunities available in other programs such as HIV, TB, and Malaria, to ensure provision of comprehensive and integrated NCDs care and treatment.


Implementation of the recommendations have been suggested to be presented during the opening ceremonies of the subsequent conference. In the most recent conference, the Permanent Secretary directed the Ministry of Health to present the implementation of these recommendations to stakeholders for monitoring purposes. Furthermore, the government has been using such recommendations to communicate important information to the public and to provide NCDs prevention and treatment services. Examples below give a summary of the news headlines after the conference: *“Serikali yatahadharisha matumizi ya Sigara, Pombe”*, which translates to ‘*The Government has warned on smoking and alcohol use, which are major risk factors for most NCDs*.’ This was the heading news in the leading daily newspaper *“Mwananchi Newspaper”* on November 10^th^, 2020. Another highlight read ‘*Government embarks on programme to curb NCDs*’, which was based on an interview in which the Chief Medical Officer stated that the government was working with scientific evidence to scale up prevention, diagnosis, and treatment of NCDs.

Advocacy through the NCD conference on the importance of insurance for all has played a key role on stimulating and developing health insurance for all Tanzanians, which is currently in discussion to be passed as a law by Tanzania’s National Assembly. In line with the recommendations to address NCDs, recommendations from the conference on re-programming the health financing for chronic diseases are currently under implementation. The national health insurance fund is undergoing major changes and the bill for universal health coverage (health insurance for all) has been tabled in Tanzania’s parliament.

The conference allowed researchers to realize the vacuum on setting NCD research priorities and prompted the idea of formulating National NCD research agenda to address the gap. The National NCDs research agenda was subsequently developed and launched in 2022. The conference also made an important effect for other commemoration events in NCDs. As an important platform for research dissemination, more than 1,000 young researchers and academicians fulfilled their potential and academic requirements through the conference.

### Challenges of organizing NCDs conferences

By and large, these conferences were a huge success. However, their preparations and organizations were not without some challenges. This limited full realization of the scope, structure, and intended targets of the conferences. The conferences faced some key challenges, the first one being limited financial support. In most of these conferences, the finances in disposition were not adequate to cater for all the requirements. Moreover, no funds were readily available for immediate use and the committee had to request funds from different organizations. This affected the timely and appropriate procurement of services. The second challenge was the conflicting schedules of the organizers. The conference organizers were voluntary members representing and working with various institutions. Competing institutional and personal schedules affected their full participation in conference preparations, causing them to miss their constructive ideas and sometimes, physical support. Another challenge was the diversity of research topics presented. In these conferences, relatively little evidence was presented on palliative medicine and NCDs-targeted innovations. This limited the opportunity to understand and analyze the local contexts, to provide recommendations to address challenges, and to increase and improve both outputs and outcomes from innovation and palliative medicine practice.

## Discussion

The Tanzania National NCDs conferences, which have now taken place for five consecutive years, have set milestones towards addressing NCDs in the country. These conferences have been organized by the Tanzanian Ministry of Health in collaboration with Muhimbili University of Health and Allied Sciences as the chief organizer working with institutions and partners.

The conferences have been providing fora for scientists in the country to convene every year to disseminate and discuss scientific evidence in the efforts to address policies and implementation gaps for NCDs response. Such scientific conferences with different agendas and themes have been organized nationally and globally with the same goal of bringing together scientists, policy makers, professionals, and other stakeholders to discuss and collaborate in important areas of science [13,14,15]. The NCDs conferences are outcomes of long processes that involve preparatory activities, identification of potential and relevant stakeholders to collaborate with, mobilization of resources (particularly funds from various organizations), request for abstracts from participants, and collection of recommendations for policy action and implementation. All processes have been essential in making the NCDs conferences impactful [16].

The NCDs conferences in Tanzania have been able to attain the intended objectives as a result of collaborative efforts of the selected subcommittees, each with specific roles and responsibilities. Similarly, effective preparations and task divisions were noted to be effective in other conferences, such as the East African Health and Scientific Conference [15]. Multi-stakeholders’ involvement and collaboration from national and international level, government, and private sectors have been embraced in other scientific conferences [15] and non-health initiatives as an important collaborative approach to attaining the goals and targets of the 2030 Agenda for Sustainable Development [17].

The organization of the National NCDs conferences would be challenging without a strong financial subcommittee and collaborative approaches to raise funds from the Ministry of health, NGOs, civil society organizations, and pharmaceutical companies. Globally, organizations such as the United States Food and Drugs Authority (FDA) have also been providing funding to support scientific meetings, conferences, and symposium owing to their importance in knowledge exchange [18]. Several funding opportunities for research conferences exist for different countries. However, the majority of these opportunities are limited to fund areas of concern impacting public health within their scope of mission [19,20]. Nevertheless, exhibition charges and participation fees have been useful to providing additional funding for the NCDs conferences and has been a common approach in other scientific conferences [14,15].

The impact of the NCDs conference has been further maximized by publishing the conference proceedings and abstracts in a journal as an issue paper [11,12]. Uploading conference presentations, posters, and abstracts to highly trafficked public repositories, and other ways of recording and sharing scientific presentations, have been highly encouraged to ensure continued sharing and dissemination of scientific knowledge [21].

The COVID-19 pandemic forced the scientific community to renovate on better ways of exchanging and transferring scientific knowledge while considering measures to combat the pandemic, such as travel restrictions and gathering bans [22]. The Tanzanian NCDs conferences were also affected and as a result, the 3^rd^ and 4^th^ NCD conferences were hybrid with both in-person attendance and virtual attendance through Zoom and YouTube livestreaming. Digital platforms have been encouraged worldwide in organizing academic conferences in the amidst the COVID-19 pandemic [22,23]. Despite geographical challenges and travel restrictions, technology has made it possible for experts to interact virtually to develop and discuss important health agendas. However, physical engagement is still recommended by some due to time differences and inequalities in accessing internet [16].

NCDs conferences provide an avenue for scientists to develop bold recommendations that catch government and stakeholders’ attention to combat NCDs. The Tanzanian NCD conferences have provided recommendations that are line with the WHO Global Action Plan for Prevention and Control of NCDs 2013–2020 based on local evidence [5]. The action plan stresses on a life course approach, empowerment of people and communities, evidence-based strategies, universal health coverage, and multisectoral action, which have been recurring themes in the Tanzania NCDs conferences and recommendations. This initiative has been impactful in changing the NCDs response and landscape in Tanzania and hence we recommended for countries with similar contexts.

## Conclusion

The burden of NCDs is rising at unprecedented rates globally and more so in the developing world. Concerted efforts of different stakeholders are needed to halt the trend of these diseases. NCDs scientific conferences provide suitable platforms where different stakeholders can share, discuss, and recommend vital strategies for addressing the contemporary situation. Engaging the right stakeholders during the conference and ensuring uptake and utilization of the recommendations is crucial for addressing the observed changes in disease epidemiology. To continue appreciating the utility of these events, prompt and adequate resource mobilization must be ensured. In turn, these will help to sustain the conference and their related impacts.

## Data Accessibility Statement

The datasets used and/or analyzed during the current study are available from the corresponding author on reasonable request.

## Additional Files

The additional files for this article can be found as follows:

10.5334/aogh.4112.s1 Supplementary Figure 1. Feedback of Conference Evaluation by the participants in 2021.

10.5334/aogh.4112.s2 Supplementary Figure 2. Stakeholders’ financial contribution to the conference.

10.5334/aogh.4112.s3 Supplementary Table 1. Country representation during NCDs Conferences.
