# Supplementary figures and images for: National Non-Communicable Diseases Conferences- A Platform to Inform Policies and Practices in Tanzania
Source: Ann Glob Health. 2024 Mar 4;90(1):18. doi: 10.5334/aogh.4112 (PMC10921961; doi:10.5334/aogh.4112)

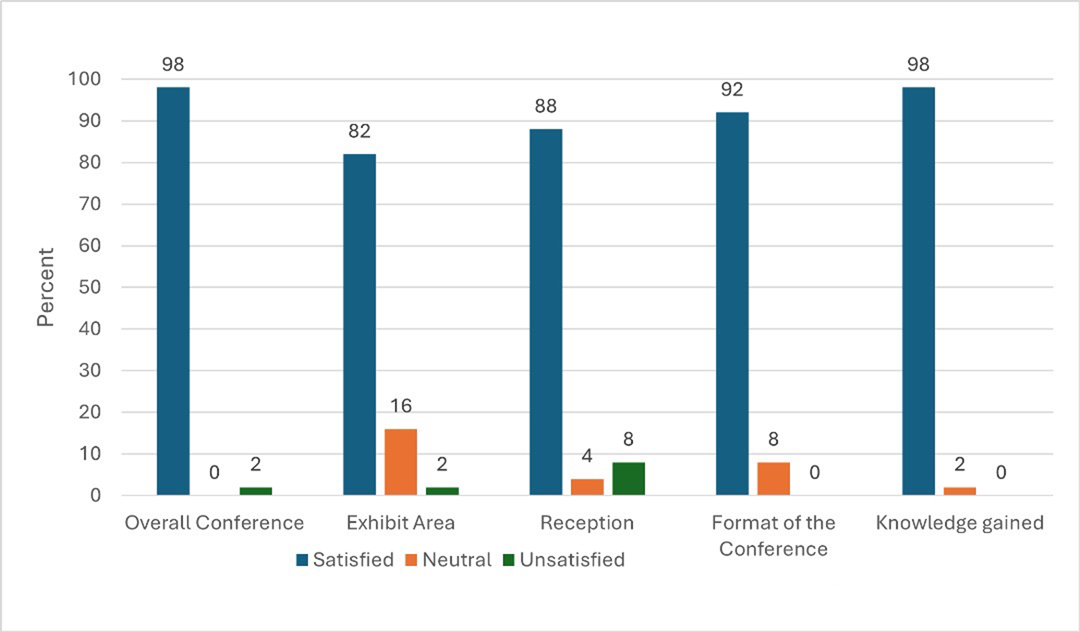

Supplement: Supplementary Figure 1. — Feedback of Conference Evaluation by the participants in 2021. [file agh-90-1-4112-s1.jpg]

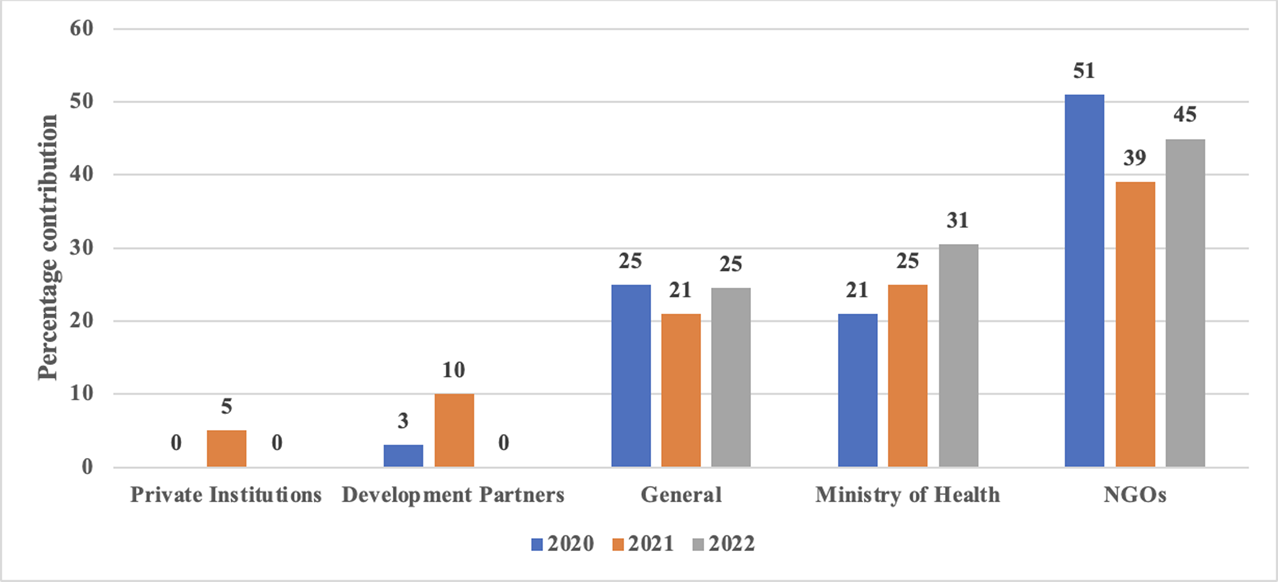

Supplement: Supplementary Figure 2. — Stakeholders’ financial contribution to the conference. [file agh-90-1-4112-s2.png]

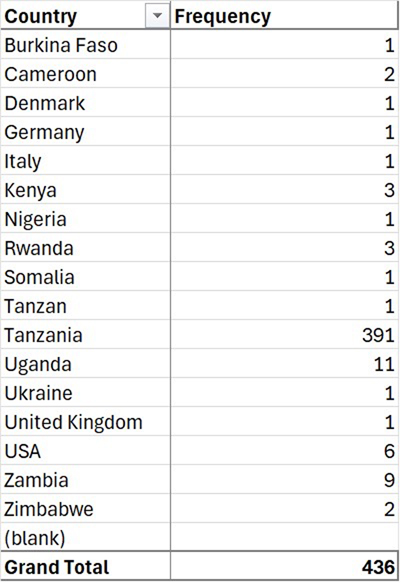

Supplement: Supplementary Table 1. — Country representation during NCDs Conferences. [file agh-90-1-4112-s3.jpg]
